# Editorial: Enhancing durability of bioelectronic implants: innovations in encapsulation materials and methods

**DOI:** 10.3389/fnins.2026.1967252

**Published:** 2026-09-11

**Authors:** Kyungjin Kim, Florian Solzbacher, Timothy G. Constandinou, Vasiliki Giagka, Loren Rieth, Adam Khalifa

**Affiliations:** 1 Department of Biomedical Engineering, University of Connecticut, Storrs, CT, United States; 2 Institute of Materials Science, University of Connecticut, Storrs, CT, United States; 3 Department of Mechanical Engineering, University of Connecticut, Storrs, CT, United States; 4 Department of Electrical and Computer Engineering, University of Utah, Salt Lake City, UT, United States; 5 Department of Materials Science and Engineering, University of Utah, Salt Lake City, UT, United States; 6 Department of Biomedical Engineering, University of Utah, Salt Lake City, UT, United States; 7 Blackrock Neurotech, Salt Lake City, UT, United States; 8 Department of Electrical and Electronic Engineering, Imperial College London, London, United Kingdom; 9 UK Dementia Research Institute, Care Research and Technology Centre, London, United Kingdom; 10 Mint Neurotechnologies Ltd., London, United Kingdom; 11 Department of Microelectronics, Faculty of Electrical Engineering, Mathematics and Computer Science, Delft University of Technology, Delft, Netherlands; 12 Department of System Integration and Interconnection Technologies, Fraunhofer-Institut für Zuverlässigkeit und Mikrointegration, Berlin, Germany; 13 Department of Mechanical, Materials and Aerospace Engineering, West Virginia University, Morgantown, WV, United States; 14 Department of Chemical and Biomedical Engineering, West Virginia University, Morgantown, WV, United States; 15 Department of Electrical and Computer Engineering, University of Florida, Gainesville, FL, United States

**Keywords:** accelerated aging, bioelectronic implants, encapsulation, flexible neural interfaces, interface adhesion, long-term reliability

Bioelectronic implants have enabled increasingly precise recording, stimulation, and modulation of physiological activity. Their continued miniaturization and transition toward thin, flexible, and mechanically compliant designs may improve close contact with neurons, tissue integration and response, and surgical accessibility (Lacour et al., 2016). However, these advantages also create substantial reliability challenges for encapsulation materials. Implanted devices are continuously exposed to water, ions, reactive species, mechanical loading and deformation, and interfacial stresses, while stimulating implants must additionally maintain stable electrodes and material interfaces under repeated electrical stimulation. Moisture penetration, hydrolysis, corrosion, oxidation, cracking, delamination, and other deteriorative reactions can progressively compromise insulation, interconnects, electrodes, and ultimately device function. Accordingly, long-term implant reliability depends not only on the intrinsic barrier properties and biostability of the encapsulation material, but also on adhesion, interface design, device geometry, fabrication quality, and the integrity of the underlying electronic components (Mariello et al., 2022). Mechanical deformation and damage to thin-film barriers can further alter their protective performance, emphasizing the need to evaluate encapsulation integrity under coupled environmental and mechanical conditions (Kim et al., 2021). For active implantable electronics, complementary strategies combining polymeric encapsulation with robust device-level and integrated-circuit design can provide additional protection against degradation in physiological environments (Nanbakhsh et al., 2025). Critical to evaluating and improving these systems are high-fidelity accelerated aging methods that better recapitulate *in vivo* conditions and enable more predictive assessment of long-term performance.

This Research Topic brings together five studies examining encapsulation and reliability across diverse implant architectures and material systems. These contributions demonstrate that long-term stability must be approached as a device-level challenge spanning material selection, structural design, manufacturing integration, failure analysis, and accelerated testing.

Niemiec et al. investigated the encapsulation of flexible polyimide microelectrode arrays using conventional atomic layer deposition (ALD) and three-dimensional atomic layer infiltration (3D-ALI). Conventional planar fabrication can leave electrode-via and device-outline sidewalls exposed, creating pathways for fluid ingress even when the major device surfaces are covered by an inorganic barrier. The proposed off-wafer 3D-ALI process conformally coated the released devices, including these vulnerable sidewalls, while producing a polymer–inorganic gradient intended to reduce mechanical mismatch. The 3D-ALI devices demonstrated improved sidewall coverage and qualitative resistance to cracking compared with conventional ALD. Nevertheless, this strategy did not significantly increase overall device lifetime under accelerated aging, as failures also originated from metal cracking, polymer–polymer delamination, and polymer–metal adhesion loss. These findings demonstrate that eliminating one permeation pathway may not extend device lifetime when other components or interfaces remain limiting.

Shah Idil et al. examined a contrasting encapsulation strategy based on medical-grade silicone rubber, plasma-enhanced adhesion, foundry passivation, and protective integrated-circuit design features. Silicone-encapsulated CMOS test structures were aged in saline at 47, 67, and 87 °C under 5V direct-current and biphasic electrical biases for as long as 4.3 real years. Despite silicone's high water permeability, no insulation failures or significant dielectric degradation were detected by electrochemical impedance spectroscopy. Instead, observed sudden failures were associated primarily with interconnection failure, including gold wire bonds on aluminum pads. This work emphasizes that effective polymer encapsulation does not necessarily require complete exclusion of water. Strong underwater adhesion prevents liquid water condensation and the formation of ionic pathways. When combined with robust passivation and careful system design, this system architecture provides long-term electrical protection in the implant environment. It also illustrates how the weakest component may shift from the encapsulation itself to interconnection technology once insulation becomes sufficiently reliable.

Nguyen et al. addressed durability under chronic *in vivo* conditions by comparing Utah electrode arrays encapsulated with existing Parylene-C layers vs. amorphous silicon carbide (a-SiC). Arrays implanted in rat motor cortex were monitored for 25 weeks using electrochemical impedance spectroscopy, cyclic voltammetry, and voltage-transient measurements. Both device types exhibited longitudinally stable impedance and maximum charge-injection trends, with their behavior becoming similar after approximately 16 weeks. The study also identified substantial differences between *in vitro* and *in vivo* electrode behavior, including changes in the open-circuit potential of the return electrode and reductions in maximum charge-injection capacity after implantation. These observations highlight the importance of evaluating encapsulation together with the electrode–tissue interface and stimulation performance rather than relying only on preimplantation measurements or a single electrochemical metric.

Jiracek et al. explored liquid-crystal polymer (LCP) as a low-moisture-uptake, thermoplastically bonded platform for thin and flexible neural interfaces. The authors integrated 500-nm-thick metallization onto embossed LCP and demonstrated reliable patterning of long, narrow traces without compromising LCP-to-LCP bond strength. Embedded structures maintained their mechanical and electrical integrity through bonding and 12 days of reactive accelerated aging at 87 °C in peroxide-containing saline. Notably, the embossed surface topography served several roles at once, strengthening the thermoplastic LCP–LCP bond, enabling thinner bonded structures, and yielding roughened platinum electrodes with lower impedance than flat counterparts. This shows that engineering topography into a thermoplastic encapsulant can simultaneously deliver fine-pitch metallization, robust bonded interfaces, and reduced device thickness.

Finally, Fluker and Judy directly examined the durability of bonding methods used between polyimide layers. Oxygen-plasma treatment produced high initial adhesion but underwent rapid, edge-driven degradation during reactive accelerated aging. In comparison, KOH/HCl treatment designed to promote polymerization reactions between the polyimide substrate and added layer produced lower initial peel strength but degraded far more slowly over the test period, yielding an estimated ≥40-fold improvement in bond lifetime. These results underscore that high initial adhesion does not necessarily predict chronic stability. Accelerated testing that incorporates reactive species, together with mechanical measurements and failure-progression analysis, can reveal degradation mechanisms that may remain undetected in conventional saline soaking.

These studies demonstrate that durable encapsulation is not defined by a single material property or test result. Long-term reliability emerges from the interaction of barrier performance, interfacial adhesion, mechanical compatibility, sidewall and edge protection, interconnect design, and manufacturing quality. The Research Topic also reinforces the need for complementary evaluation methods, including accelerated aging, reactive aging, mechanical testing, electrochemical monitoring, failure-mode analysis, and chronic *in vivo* validation. All these constitute a necessary foundation for better understanding and improving encapsulation failure modes which will ultimately enable far longer safe and functional implantation times than currently possible, up to true lifetime implants. We thank all authors and reviewers for their contributions and hope that this Research Topic supports the development of more predictive reliability methods and more robust, clinically translatable bioelectronic implants.
